# SCFIA: a statistical corresponding feature identification algorithm for LC/MS

**DOI:** 10.1186/1471-2105-12-439

**Published:** 2011-11-11

**Authors:** Jian Cui, Xuepo Ma, Long Chen, Jianqiu Zhang

**Affiliations:** 1 Department of Electrical and Computer Engineering, the University of Texas at San Antonio, One UTSA Circle, San Antonio, TX 78249, USA

## Abstract

**Background:**

Identifying corresponding features (LC peaks registered by identical peptides) in multiple Liquid Chromatography/Mass Spectrometry (LC-MS) datasets plays a crucial role in the analysis of complex peptide or protein mixtures. Warping functions are commonly used to correct the mean of elution time shifts among LC-MS datasets, which cannot resolve the ambiguity of corresponding feature identification since elution time shifts are random. We propose a Statistical Corresponding Feature Identification Algorithm(SCFIA) based on both elution time shifts and peak shape correlations between corresponding features. SCFIA first trains a set of statistical models, and then, all candidate corresponding features are scored by the statistical models to find the maximum likelihood solution.

**Results:**

We test SCFIA on publicly available datasets. We first compare its performance with that of warping function based methods, and the results show significant improvements. The performance of SCFIA on replicates datasets and fractionated datasets is also evaluated. In both cases, the accuracy is above 90%, which is near optimal. Finally the coverage of SCFIA is evaluated, and it is shown that SCFIA can find corresponding features in multiple datasets for over 90% peptides identified by Tandem MS.

**Conclusions:**

SCFIA can be used for accurate corresponding feature identification in LC-MS. We have shown that peak shape correlation can be used effectively for improving the accuracy. SCFIA provides high coverage in corresponding feature identification in multiple datasets, which serves the basis for integrating multiple LC-MS measurements for accurate peptide quantification.

## Background

Liquid Chromatography-Mass Spectrometry/Tandem Mass Spectrometry (LC-MS/MS) is a powerful tool for protein identification and quantification [1]. One important task in LC-MS/MS processing is the identification of corresponding features (peaks registered by identical peptides) in multiple datasets, which is critical for the integration of quantification information to reduce measurement variation [2].

Before other discussions, we first introduce some definitions that are used throughout the paper. A feature is the two dimensional (retention/elution time - m/z) signal registered by a single charge variant of a peptide. When we consider extracted-ion-chromatograms (XICs), a feature is represented by its LC elution peak in an LC-MS/MS run. If a peptide is picked up by Tandem MS, then its LC elution peak can be located exactly in LC-MS. We refer to such LC peaks as "features with identity". If a peptide is not picked up by Tandem MS, then its elution peak location would be unknown, and its LC peak is called "a feature with unknown identity".

If several datasets are collected in an experiment, then each dataset has an associated list of Tandem MS identified peptides. We simply refer to the peptides associated with a dataset Q1, for example, as Q1 peptides. The union of all peptides from all datasets is noted as the "union peptide set". When corresponding features of a peptide is found in all datasets, we say that the peptide is "completely identified for quantification", or simply "completely identified/quantified" in different context.

Current alignment approaches focus on correcting the mean of elution time shifts between datasets using warping functions. Warping function based methods can be categorized as profile- or feature-based. Profile-based approaches align total-ion-chromatograms (TIC) or higher-resolution profiles based on the full, unprocessed data obtained in LC-MS experiments. The most basic profile-based methods compare the difference in the TICs [3]. A method called correlation optimized warping (*COW*) was proposed by Nielsen [4]. Bylund proposed many modifications to *COW *[5]. Parametric time warping (*PTW*) was proposed by Eilers [6]. Van showed an extension of PTW called semi-parametric time warping (*STW*) [7]. Prince generated the warping function based on dynamic time warping with a one-to-one (bijective) smooth warp-function called Obi-warp [8].

Feature-based approaches focus on either aligning chromatogram peaks, aligning features or significant features in images [9,10]. In an initial feature detection step, these approaches try to distinguish relevant features of peptides and irrelevant noise in the data. Among these methods, a very sophisticated algorithm called LCMSWARP has been published by Jaitly [11]. Another paper [12] compared six freely available alignment algorithms, and found that OpenMS [13] performs the best on both proteomics and metabolomics data. Most recently, Voss [14] proposed a method which combines hierarchical pairwise correspondence estimation with simultaneous alignment and global retention time correction. Voss's paper focuses on the alignment of multiple datasets at the same time. However, the performance is slightly worse than that of OpenMS on proteomics data.

**Figure 1:**
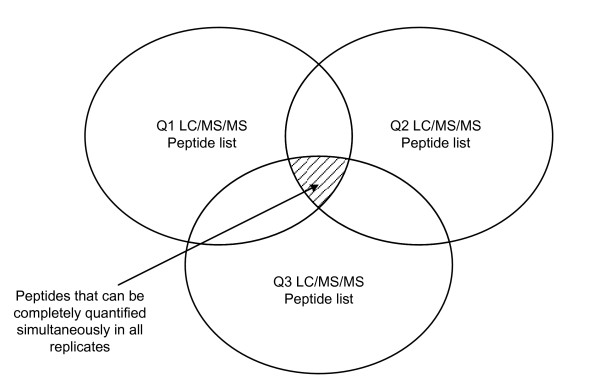
**Venn-diagram of Tandem MS identified peptides in three LC-MS/MS datasets**. Typically, only a small overlap exists among peptides identified in different datasets, which means that only a small fraction of identified peptides can be completely quantified if the usual processing procedure is employed.

In LC-MS/MS, shorter elution time, which leads to crowded XICs, is often desirable for increasing the throughput because it cuts down experimental time [15]. In such cases, there could be multiple elution peaks within a narrow elution time window after warping function correction, and it is ambiguous which peaks are corresponding. We have observed in some cases that the nearest LC peak to the warped time point is not the real corresponding one, and warping function based methods have a limitation in improving alignment accuracy. In addition, some popular alignment algorithms, such as OpenMS [13] or msInspect [16], are designed to work in a procedure that results in low quantification coverage [17], which can be summarized as the following: 1. Perform LC-MS peak identification in each dataset; 2. Perform alignment and corresponding feature identification; 3. Perform Tandem MS peptide identification; and 4. Link Tandem MS identified peptides to aligned corresponding features. Generally, only a small overlap exists between them, and only a small portion of identified peptides can be completely quantified. MaxQuant [18] improves quantification coverage greatly by performing an extra step that looks for the LC elution peaks of identified peptides in LC/MS. In this way, almost all identified peptides can be quantified at least once. But still, complete quantification coverage is limited to the intersection of Tandem MS identified peptides, which is expected to be small since Tandem MS picks up peptides randomly. This situation is shown in Figure 1.

**Figure 2:**
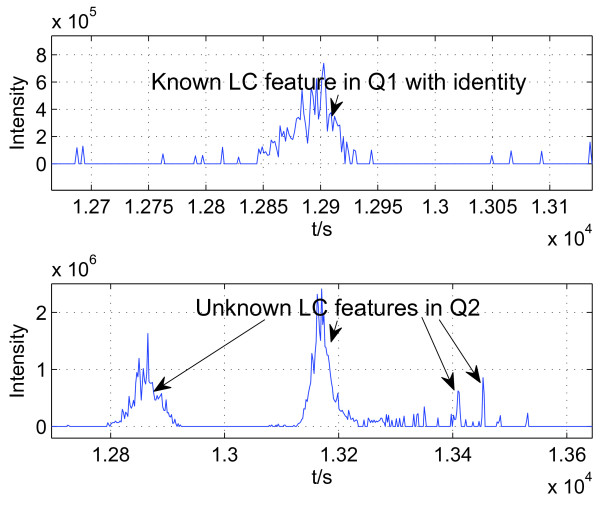
**Illustration of the problem considered in SCFIA**. The sequence information and the elution time of a peptide in Q1 is known based on Tandem MS identification information on the top panel. On the bottom panel, we are not sure which elution peak is the corresponding feature of the peptide of interest in Q2 due to random elution time shifts.

Given a small intersection between peptide lists, we know that the union of the lists must be significantly larger. If most peptides in the union set can be completely identified in all datasets, then complete quantification coverage can be improved significantly. To this goal, given a list of Q1 peptides with identity, we consider the problem of finding their corresponding features in dataset Q2. This problem is illustrated in Figure 2. Once this problem is solved, complete identification is possible for every peptide in the union set, which has identity in at least one dataset that can be treated as Q1, and any remaining dataset can be treated as Q2.

To address the proposed problem, we develop a Statistical Corresponding Feature Identification Algorithm (SCFIA) which identifies corresponding features not only based on matching elution times but also elution peak shapes. We build statistical models which can be used to evaluate the probability of candidate feature pairs as corresponding ones. The identification of corresponding features can be applied to various LC-MS datasets under different experimental conditions without user supplied information. Testing results show that SCFIA improves accuracy and complete quantification coverage significantly.

The proposed algorithm is designed for instruments with high mass resolution which have very few overlapping LC elution peaks within XICs. For example, with a mass resolution of 60, 000*FWHM *on a Orbitrap instrument, there are very few overlapping elution peaks using a mass window of 10 parts-per-million (ppm) for extracting XICs, and the proposed algorithm can be applied. Such a resolution and mass accuracy is routinely available nowadays.

### Datasets

We test and develop SCFIA based on freely available datasets, which can be downloaded from https://proteomecommons.org/dataset.jsp?i=74476. Group1 datasets are

1. 20090608_Orbi6_TaGe_SA_TUMOR_5mix1_01.raw (Group1 Q1)

2. 20090608_Orbi6_TaGe_SA_TUMOR_5mix1_02.raw (Group1 Q2)

3. 20090608_Orbi6_TaGe_SA_TUMOR_5mix1_03.raw (Group1 Q3)

Group2 datasets are:

1. 200090815_Velos5_TaGe_SA_Silacmix_TOP15_01.raw(Group2 Q1)

2. 200090815_Velos5_TaGe_SA_Silacmix_TOP15_01.raw(Group2 Q2)

3. 200090815_Velos5_TaGe_SA_Silacmix_TOP15_01.raw(Group2 Q3)

Group1 represents data from three fractions of breast cancer tissue together with a super-SILAC mix collected on an Orbitrap instrument. Group2 represents three technical replicates without prior separation collected on a new generation LTQ-Orbitrap Velos instrument. These two groups are representative of real biological datasets collected on different instruments, each of which contains hundreds of thousands of isotopically labeled peptides in appropriate amounts. For more information about super-SILAC data, please check the original paper [19]. We observe that

1. the warping function is non-linear; and

2. the elution peaks are crowded.

Comparing to the simple protein mix datasets in [12], where LC elution peaks are sparse, the super-SILAC datasets are more complex in protein composition, which lead to crowded XICs because many peptides with similar masses are eluted out within a short period of time. If we only correct the mean of time shifts between corresponding features, many peaks will be wrongly matched because there are LC peaks in the close vicinity of the true corresponding ones.

## Methods

### Tandem peptide identification

We use X!Tandem [20] in Trans-Proteomic Pipeline (TPP) [21] and MaxQuant for Tandem MS identification. In both TPP and X!Tandem, we select the International Protein Index(IPI)-human database version 3.68 as the source of protein sequences. In the TPP, X!Tandem with Kscore is applied as the search engine. Parent mass and fragment ions are searched with maximal mass errors of 7ppm and 0.5 Dalton respectively. Methionine oxidation and N-terminal acetylation are considered as variable modifications and cysteine carbamidomethylation is selected as the fixed modification. SILAC labeling is also considered as a variable modification. In the analysis, the minimum length of peptides is set to 6, and the maximum number of missed cleavage sites is set to 2. Finally PeptideProphet [22] of TPP is used to validate the search results, and peptides are annotated with PeptideProphet true positive probabilities (short noted as PeptideProphet probabilities). MaxQuant (version 1.1.1.25) is used with the same settings as that of the X!Tandem, except that the validation step is done by the decoy method. The IPI human database is decoyed by Andromeda [23], and the false discovery rate is set to 0.01.

### Ground truth list generation

To test SCFIA and train statistical models, we will need a ground truth list that contains peptide identification and elution time information in both Q1 and Q2. The ground truth list shall contain truly existing peptides through reliable identification. However, it is impossible to get a "pure" ground truth list. Tandem MS spectra are affected by interfering ions and thermal noise. There are some falsely identified ones in the reported peptide list. We can apply different PeptideProphet probability thresholds to control the false positive rate of the ground truth list. With these considerations in mind, we select the ground truth list in the following procedure:

1. In each dataset, select one retention time for each unique peptide identification. Sometimes, we find that a unique peptide is identified multiple times. In such cases, we pick the identification with the highest PeptideProphet probability.

2. We filter peptides by applying a PeptideProphet probability threshold.

3. We select peptides that are identified in both Q1 and Q2 to form the ground truth list with information of retention_time_sec, m/z value, and peptide sequence.

The ground truth list is further divided to a training and a testing set. The training set is used for statistical model training and the testing set is used for performance evaluation. Since features with higher intensities are less corrupted by noise, features with top 20% intensities are selected to form the training set. In Q1 and Q2 datasets from Group1, the training set contains 270 peptides, and the testing set contains 1425 peptides, which are annotated with their retention times in both Q1 and Q2.

Note that a pair of non-corresponding features can be obtained by replacing one of the features in a corresponding pair with a random feature from the same XIC of the replaced one. In this way, we can construct a non-corresponding feature training set.

Note that the higher the PeptideProphet probability threshold is, the purer the ground truth list. The threshold will affect the calculated accuracy in corresponding feature identification. For example, at a threshold of 95%, at least around 5% of the testing peptides are false positives, which cannot be matched to their LC peak intervals recorded in the ground truth. Consequently, the calculated corresponding feature identification accuracy can not exceed 95% significantly.

In contrast to the ground truth list selection process in [12], we do not filter features based on retention time to avoid introducing bias to the training set. The "pureness" of the ground truth list is controlled by the threshold on PeptideProphet probability. The threshold can be raised to reduce the number of outliers.

### Performance evaluation based on the testing set

Before we describe the algorithm, we want to clarify the performance evaluation method used in this paper. After we get the testing set, we pretend that we know the identities of the testing peptides in Q1, but not in Q2. We then apply SCFIA. If an identified corresponding feature has an elution time that differs from what has been recorded in the ground truth of Q2, then an error is registered. Finally, we calculate accuracy as the ratio between the total number of correctly identified corresponding pairs over the total number of peptides in the testing set. Note that this accuracy measurement is equivalent to the precision rate in [12] when considering pair-wise alignment.

In SCFIA, we first use the training set to construct the statistical models. We then evaluate the performance based on the testing set. Finally, we compare the performance SCFIA to that of OpenMs (which is described as the best [12]), and a warping function based method (Gwarping).

### Statistical corresponding feature identification algorithm (SCFIA)

**Figure 3:**
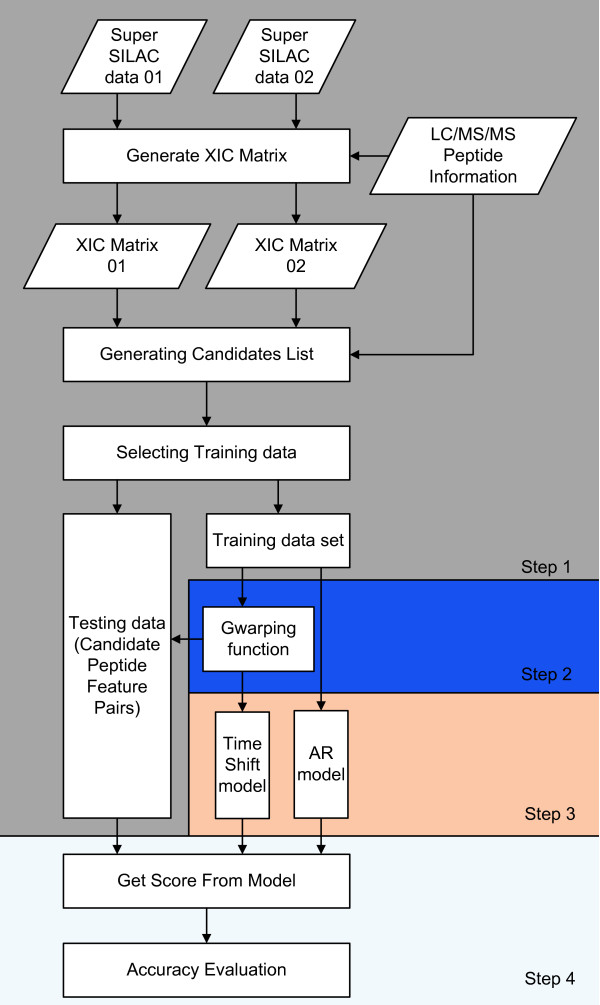
**Flow diagram of the SCFIA algorithm**.

SCFIA aims at evaluating the probability that a given pair of peptide features are corresponding. The algorithm has four processing steps: 1.) Pre-processing aims at identifying a set of initial LC elution peaks as corresponding feature candidates; 2.) Mean time shift correction between Q1 and Q2. This is achieved by estimating a warping function based on the training set; 3). Training of statistical models for corresponding feature identification; and 4). Evaluating the likelihood probability of all candidate corresponding features in Q2 given an LC peak in Q1. The candidate with the highest likelihood probability will be selected as the corresponding feature. The flow diagram of SCFIA is shown in Figure 3. The details of the algorithm is as the following:

#### Step 1: Pre-processing of LC/MS data

Preprocessing aims at finding LC elution peaks of Q1 peptides with known identity, and finding their corresponding feature candidates in Q2 where their elution intervals are unknown. The following processing steps are performed.

1. To identify possible LC peak intervals for a given peptide in both Q1 and Q2, we first calculate its XICs at its mono- and first isotope m/z values in the charge state that it has been identified in Tandem MS.

2. We use the XIC at the higher isotope position to detect up to *n *high intensity regions by applying a threshold at three times the background noise standard deviation above the median noise level. Only one interval corresponds to the elution interval of the peptide. In Q1, the exact interval is known by selecting the interval that includes the retention_time_sec recorded in the ground truth.

3. In Q2, we employ the same process as that in Q1. However, without identification information, the exact elution interval is unknown, and we treat all detected intervals as corresponding feature candidates, which should include the true corresponding one.

Given an identified LC peak interval in Q1, there are the *n *candidates in Q2, which form *n *candidate corresponding feature pairs.

#### Step 2: Mean elution time shift correction

**Figure 4:**
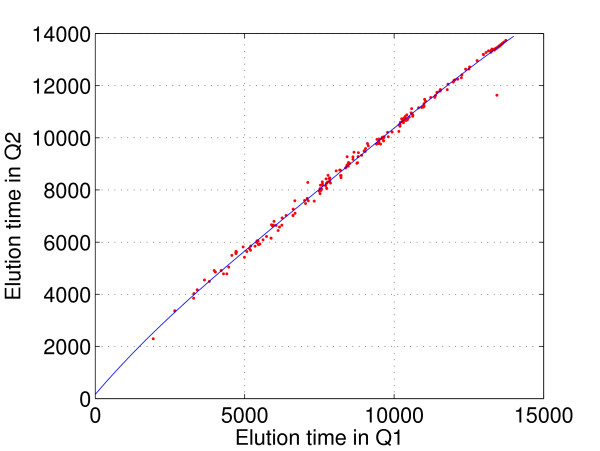
**The Gwarping function between Q1 and Q2 in super-SILAC dataset Group1**. Although the Gwarping function (solid line) is derived based on 270 corresponding feature elution time pairs in the training set, it fits the rest of 1425 time pairs (dots around the warping function) in the testing set very well. The warping function is not a linear function.

The mean time shifts between corresponding features can be corrected using a warping function. In the past, numerous algorithms have been developed for finding warping functions [3,9-11]. However, these algorithms seldom use elution time information reported by Tandem MS for estimating the warping function except those in Jaitly [11] and Palmblad [24]. However, nowadays, with much higher coverage in Tandem MS, a list of true elution time shifts is almost always available. In our study, the training ground truth list is annotated with elution time values in both Q1 and Q2, and we can simply use the Matlab function *polyfit*(·) to estimate the warping function by regressing the elution time points in Q2 to those in Q1. This generates a very good estimation of the mean of time shifts as shown in Figure 4. Note that this simple warping function can be non-linear, and it is referred as the Ground-Truth based warping (Gwarping) function. The Gwarping function differs little if we use more than 200 time points.

To evaluate the performance of Gwarping, we first use the Gwarping function for mean time shift correction, then we assign the nearest features in Q2 as corresponding ones. We find that the alignment performance of Gwarping exceeds that of OpenMS, which is considered as the best in [12]. This suggests that warping based on Tandem MS identification is reliable. Due to this reason, we use Gwarping as a representative warping function based method to compare with SCFIA.

#### Step 3: SCFIA models

In the third step, we build statistical models of corresponding features.

##### Parameters considered in the model

After pre-processing both Q1 and Q2, we obtain a training set of corresponding features, based on which, we can train our statistical models. The parameters considered are elution time shift and LC peak shape correlation between corresponding features. These two parameters are independent. Elution time shift is mainly affected by varying experimental conditions, and LC peak shape depends on the physicochemical characteristics of a peptide.

Elution time shift has been used as the most important parameter for LC peak alignment traditionally. In SCFIA, the time shift is assumed to have a Gaussian distribution [25] after mean correction, whose parameters can be estimated from the training set.

LC peak shape of peptides is another important parameter. Under similar experimental conditions, identical peptides form similar LC peaks, while different peptides form different LC peak shapes. Similarity between two LC peaks can be measured by the *R*^2 ^statistics, which indicates how well a regression line approximates the observed data points. An *R*^2 ^of 1.0 means that the regression line perfectly fits the data, while 0 means the poorest fit. For details please see [26]. When we regress an LC peak in Q1 to one in Q2, the resulted *R*^2 ^statistic is noted as the alignment *R*^2^(*AR*) statistics. ARs can be calculated using the Matlab function *regress*(·).

Two statistical models can be built for these two parameters. Suppose that *AT *is the elution time shift between two peptide peaks, we can write

(1) P(y)=P(AT)P(AR),

where *P*(**y**) represents the probability that the considered feature pair with time shift *AT *and peak shape correlation *AR *is corresponding. Both *P*(*AT*) and *P*(*AR*) are given by the statistical models we constructed from the training set. Our goal is to find the corresponding feature pair that maximizes the likelihood probability function in (1).

##### Elution time shift model

In Figure 4, we plot the warping function estimated from elution time shifts of corresponding features from the training set of Q1 and Q2 from Group1. After applying the warping function, we calculate the remaining time shifts between corresponding features.

In the past, the time shifts are assumed as Gaussian [11], and we can write *P*(*AT *| *μ*, *σ*^2^) ~ *N*(*μ*, *σ*^2^), where *μ *represents the mean and *σ*^2 ^the variance. We estimate (*μ*, *σ*^2^) from the remaining time shifts between corresponding features using the Matlab function *normfit*(·).

**Figure 5:**
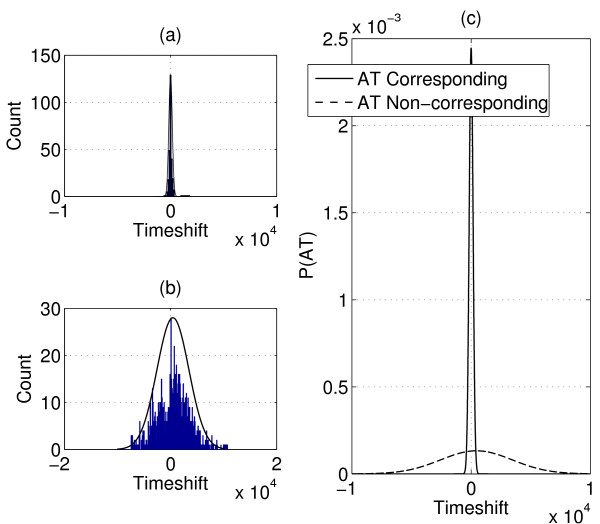
**Statistical model of elution time shifts after applying the Gwarping function**. (a) Normalized histogram and fitted model of AT between corresponding features. (b) Normalized histogram and fitted model of AT between non-corresponding features. (c) AT statistical models of corresponding and non-corresponding features.

We plot the histogram of AT (after mean time shift correction) together with its estimated statistical model for corresponding features in Figure 5(a). We also plot the normalized histogram and the fitted model of non-corresponding features in Figure 5(b). In Figure 5(c), we plot the two statistical models together, and we can see that there is a big difference in the distribution of AT, which allows us to differentiate corresponding and non-corresponding features. The fitted model of AT between corresponding features is then substituted as *P*(*AT*) in (1).

##### AR statistic model

**Figure 6:**
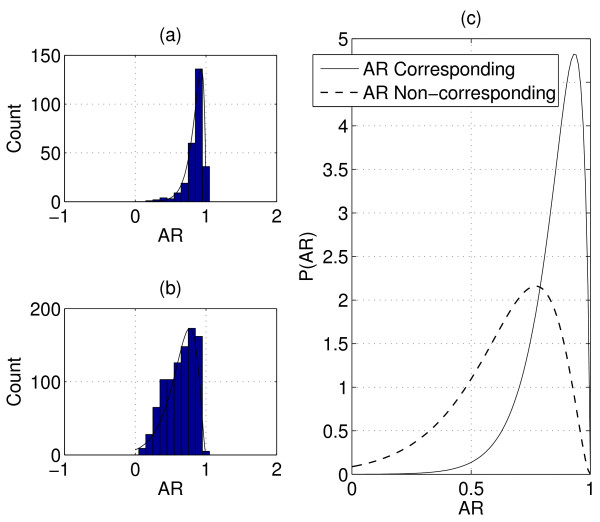
**Statistical model of peak shape correlations**. (a) Normalized histogram and fitted model of AR between corresponding features. (b) Normalized histogram and fitted model of AR between non-corresponding features. (c) AR statistical models of corresponding and non-corresponding features.

To find a suitable model for *AR*, we plot the normalized histogram of *AR *between corresponding features in the training set of Q1 and Q2 from Group1. In Figure 6(a), we can see that most AR values are around 0.85 between corresponding features. Let *X *= 1 - *AR*, and we model × as a random variable that follows the gamma distribution, *X *~ *Gamma*(*k*, *θ*). We can write

(2) $$f\left(x|k,\theta \right)=x^{k-1}\frac{exp\text{(}-\frac{x}{\theta }\text{)}}{\theta^{k}\Gamma \left(k\right)},$$

where *k *and *θ *are parameters of the Gamma distribution, which can be estimated using the Matlab function *gamfit*(·). In Figure 6(a), we plot the fitted Gamma distribution with the normalized histogram of AR for corresponding features. The normalized histogram and fitted model of AR for non-corresponding features are plotted in Figure 6(b). In Figure 6(c), we compare the difference in fitted distributions of AR between corresponding and non-corresponding features. We can see a notable difference in this example. Note that Group1 is composed of datasets from different peptide fractions, thus there exist significant concentration variations which do not lead to significant deterioration of peak shape correlations between corresponding features. This indicates that AR is a valuable parameter for corresponding feature detection. The fitted Gamma distribution is then used as *P*(*AR*) in (1).

#### Step 4: Estimate probabilities of candidate corresponding feature pairs

In the fourth step, between any pair of candidate features, we first calculate its AT and AR, which are plugged in (1) subsequently. The candidate pair with the highest likelihood probability will be reported as the corresponding one.

**Figure 7:**
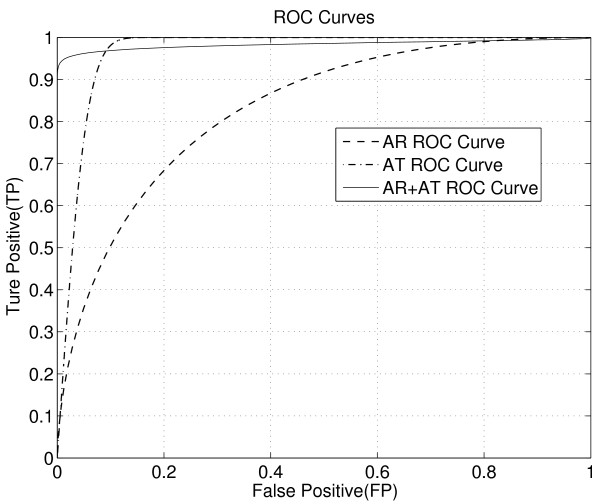
**ROC Curves of AT, AR and AT+AR (the combined probability score)**. We can see that by using the combined probability score, the ROC is the best on regions with low false positive rate.

Based on fitted distributions of AT and AR for corresponding and non-corresponding features, we can plot their Receiver Operating Characteristic (ROC) curves. In Figure 7, we plot the ROC curves of AT, AR, and the combined probability score as calculated in (1). We can see that the combined probability score is expected to give the best performance when the False positive rate is below 8%. This predicts that using both AT and AR will provide performance gain.

## Results

### Accuracy in corresponding feature detection

To compare the performance of SCFIA with other methods, we use the testing ground truth list of Q1 and Q2 from Group1. We apply various algorithms for corresponding feature identification. When applying SCFIA, we use a PeptideProphet probability threshold of 95% and a 10*ppm *mass window for calculating the XICs. This mass window is selected based on the mass accuracy of the instrument, which should be adjusted for different instruments.

#### Comparison with OpenMS

OpenMS(Version 1.7.0) is evaluated to have the best performance in [12]. The details of the simulation process of OpenMS can be found in the [Additional file 1]. We set RT to two possible values, 500 and 700, while MZ is set as 0.01. We try different settings to ensure the best result. OpenMS achieves a 79.79% and 80.35% accuracy under two different settings which have little difference.

#### Comparison with Gwarping

To estimate the improvement of SCFIA over warping function based methods, we want to compare the performance of SCFIA to Gwarping. After applying the Gwarping function, the elution time of each peptide in Q1 is mapped to Q2, then the LC peak which is the closest to the mapped time point is considered as the detected corresponding feature in Q2. By employing this simple method, the accuracy is 89.89%, which is higher than that of OpenMS. This result is not surprising because OpenMS does not consider non-linear warping functions. There are a total of 144 peptides that are not aligned correctly out of 1425 testing peptides. We inspect manually and find that these peptides have interfering LC peaks that are closer to the mapped time points than the true corresponding ones in Q2. The proportion of such peptides strongly depends on experimental settings. If shorter elution time is desired, then more peptides will have close neighbors, and warping function based methods will be less effective in finding corresponding features.

#### Performance of SCFIA

In SCFIA, we detect corresponding features not only based on AT but also on AR. The result of our algorithm is summarized in Table 1. SCFIA achieves the highest accuracy of 94.18% among the three algorithms tested. Out of 144 peptides that Gwarping can not align, 95 are correctly aligned by SCFIA. In [Additional file 1], we show an example of a peptide which is not aligned correctly by Gwarping, but aligned correctly by SCFIA. In that example, there is a nearly 50 fold difference in LC peak height, yet the peak shape correlation is still high. This indicates that peak shape correlation stands up pretty well even when there are significant concentration variations.

**Table 1:** Corresponding feature identification accuracy

Testing set of Q1 and Q2 from Group1			
**Algorithm**	**SCFIA**	**Openms**	**Gwarping**

Accuracy	94.18%	80.35%	89.89%

We manually inspect the 49 peptides that are not aligned by SCFIA. We find that for these 49 peptides, their corresponding features specified by the ground truth do not agree in elution time and peak shape as well as interfering ones. We show an example in [Additional file 1]. We suspect that these peptides are false positives in Tandem MS identification. If this assumption is true, we should be able to observe an increased accuracy rate as we raised the threshold on PeptideProphet probability.

We test this hypothesis by increasing the PeptideProphet probability threshold, and the results are summarized in Table 2. We can see that corresponding feature identification accuracy closely follows the threshold. This suggests that SCFIA can match nearly every true positives in the "ground truth" list, and its performance is near optimal.

**Table 2:** Corresponding feature identification accuracy v.s. PeptideProphet probability threshold

Testing set of Q1 and Q2 from Group1		
**PeptideProphet probability**	**No. of peptides tested**	**Accuracy of SCFIA**

95%	1425	94.18%

98%	1252	95.13%

99.9%	210	97.62%

99.99%	23	100%

We have also tested the accuracy of SCFIA between the remaining data pairs in Group1 and Group2. The results are also summarized in Table 3. Group1 is composed of LC/MS datasets from different fractions where the variations in concentration and elution time are larger than that between replicates in Group2. In Table 3, we can see that SCFIA consistently provides performance gain by using the combined probability score in Group1. In contrast, for Group2, the performance of using AT alone is already very close to the optimal, and using the combined probability score provides a small gain in two out of the three cases. This phenomenon can be attributed to the smaller elution time variations between technical replicates. This shows that SCFIA is more effective in corresponding feature identification when there are high elution time and concentration variations.

**Table 3:** Corresponding feature identification accuracy

Testing set of different dataset pairs						
**Data Pairs**	**Q1 Q2 Grp.1**	**Q2 Q3 Grp.1**	**Q1 Q3 Grp.1**	**Q1 Q2 Grp.2**	**Q2 Q3 Grp.2**	**Q1 Q3 Grp.2**

Accuracy AT	89.89%	86.05%	87.08%	95.08%	93.05%	96.73%

Accuracy AR	70.70%	69.15%	62.46%	72.83%	70.76%	70.83%

Accuracy AT+AR	94.18%	92.07%	89.85%	94.96%	95.01%	97.98%

### Complete quantification coverage

**Figure 8:**
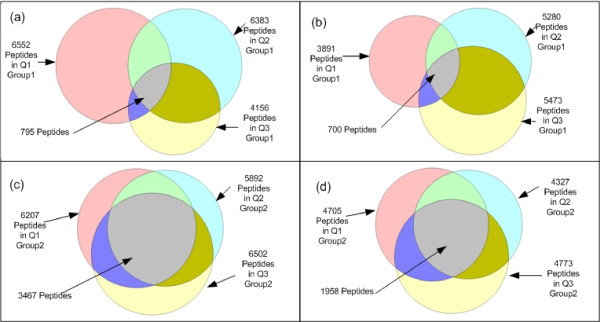
**Number of peptides identified in different data groups when using different Tandem MS search engines**. (a): Number of peptides in Group1 (X!Tandem). (b): Number of peptides in Group1 (MaxQuant). (c): Number of peptides in Group2 (X!Tandem). (d): Number of peptides in Group2 (MaxQuant). We can see that the intersection is very small comparing to the union in all cases.

SCFIA is designed for the complete quantification of the union peptide set. We first investigate Group1 datasets (Q1, Q2, Q3) from three fractions. After pre-processing of Tandem MS scans using X!Tandem and TPP, we obtain a list of Tandem MS identified peptides. Then we combine peptides with identical identifications, and filter out peptides with PeptideProphet probability less than 0.95. In Figure 8(a), we illustrate the Venn-diagram of the sizes of Tandem MS identified peptide lists. We can see that the overlap between any two fractions is quite small, and the size of the union (a total of 12874) is significantly larger than that of the intersection (795).

We employ the following procedure for the complete identification of all peptides in the union set. We first select Q1 peptides, and find their corresponding features in Q2 if their identities are unknown. Then the same procedure is repeated from Q1 to Q3. Subsequently, we focus on Q2 peptides, and we find all corresponding features in Q1 and Q3 if their identities are not known yet. Lastly, we focus on Q3 peptides. This procedure is repeated for all peptides in all datasets with unknown identities until complete identification. Using this procedure, a total of 11124 (93.78%) peptides are completely identified in all three datasets, 590 are identified in at least two datasets.

We then investigate Group2 datasets (Q1, Q2, Q3) from three technical replicates. The PeptideProphet probability threshold we choose is still 0.95. In Figure 8(c), we show that the union has 9239 peptides, and the intersection has 3467. For testing purposes, peptides detected in different charge states and different datasets are removed from the list, which leaves 5628 peptides without complete identifications. Using SCFIA, a total of 5655 (99.52%) peptides are completely identified in all three datasets, and 22 are identified in at least two datasets. Since Group2 datasets are from replicates, a higher complete identification rate is expected than that of Group1.

With complete identification, these peptides can be quantified completely. Since peptide quantification is a lengthy topic, we leave it out of this paper.

#### Comparison with MaxQuant

MaxQuant [18] is a popular tool that provides both Tandem MS identification and quantification. We want to compare the peak identification coverage of SCFIA with that of MaxQuant. To this end, we employ MaxQuant (Version 1.1.1.25) to process super-SILAC datasets Q1, Q2, and Q3 in Group1 and Group2. The size of peptide identification results is summarized in the Venn-diagram in Figure 8(b). We can see that the union set of Group1 contains a total of 10511 peptides, and the intersection between them is 700. Thus based on Tandem MS identification information, only 556 peptides can be completely quantified in all three datasets. In contrast, after applying SCFIA, a total of 8938 peptides are identified in all three datasets in the first group.

The same process is repeated in Group2, and the results are reported in In Figure 8(d). Significant advantage of SCFIA is reported again.

The results on elution peak identification coverage using MaxQuant and X!tandem are summarized in Table 4. These results show that under different Tandem MS search engines and different sample compositions, the intersection set is always pretty small comparing to the union set. SCFIA is very effective in improving complete identification coverage, based on which, accurate quantification can be performed for nearly all identified peptides.

**Table 4:** Complete identification coverage

Complete Identification Coverage				
	**Xtendem (Data Group1)**	**Maxquant (Data Group1)**	**Xtendem (Data Group2)**	**Maxquant (Data Group2)**

Q1 ∪ Q2 ∪ Q3- Q1 ∩ Q2 ∩ Q3 peptides analyzed by SCIFA	11862	9582	5682	5463

SCFIA identified completely	11124	8938	5655	5432

SCFIA identification coverage	93.78%	93.28%	99.52%	99.43%

## Discussion

Through testing, we can see that SCFIA can be applied in the alignment of both technical replicates and datasets collected from different LC/MS runs.

The accuracy results based on Group1 and Group2 data suggest that SCFIA is more effective when there are high elution time and concentration variations. In such cases, using peak shape correlation improves the performance. However, the improvement changes with experimental conditions. When elution time variation is small, and there exists long gaps between elution peaks, then alignment based on elution time is sufficient. However, when elution time variation is large, and gaps between LC peaks are small, peak shape correlation becomes useful in performance improvement. Users can always decide if using peak shape correlation will provide performance gain by inspecting the ROC curves estimated by SCFIA. In experiments where peak shape reproducibility is not strong, or when the XICs are not crowded, then it may be sufficient or necessary to use AT alone.

SCFIA requires a number of "common" identifications for training the statistical models. Generally the more common identifications the better. Preferably, there are around 200 common identifications. We observe no obvious difference in performance in our experiments when the size of the training set increases beyond this number.

## Conclusion

In this paper, we propose a new Statistical Corresponding Feature Identification Algorithm (SCFIA) for the identification of corresponding features in different LC-MS/MS datasets. The main innovation of the algorithm is the use of statistical models for both elution time shifts and peak shape correlations, which provides maximum likelihood estimates of corresponding features. The algorithm allows accurate corresponding feature identification with crowded elution profiles. We verify the algorithm on two groups of super-SILAC datasets, and the performance is shown to be better than warping function based methods including OpenMS. SCFIA is shown to have very high accuracy in corresponding feature identification and the performance is near optimal.

SCFIA can be utilized for the complete identification of elution peak intervals of Tandem MS identified peptides in multiple datasets. We have verified that SCFIA provides high coverage in complete identification which will lead to more accurate quantification in differential analysis for biomarker discovery.

## Availability and Requirements

Project name: SCFIA project; Operating system(s): Windows XP/vista/7; Programming language: Matlab; Licence: GNU GPL; Any restrictions to use by non-academics: licence needed. The related material including the testing dataset can be found at the project webpage http://compgenomics.utsa.edu/SCFIA.html.

## Authors' contributions

JC developed and tested the algorithm, wrote the paper. XM and LC provided assistance in data interpretation. JZ conceived the idea, advised on the development of the algorithm and revised the paper. All authors have read and approved the final manuscript.

## Supplementary Material

Additional file 1 **Supplementary Information**. In this file we provide supplementary information. Click here for file
