# Supplementary material for: SCFIA: a statistical corresponding feature identification algorithm for LC/MS
Source: BMC Bioinformatics. 2011 Nov 11;12:439. doi: 10.1186/1471-2105-12-439 (PMC3233610; doi:10.1186/1471-2105-12-439)
Supplement: Additional file 1 — Supplementary Information. In this file we provide supplementary information. [file 1471-2105-12-439-S1.PDF]

SCFIA: A Statistical Corresponding Feature Identification Algorithm for LC/MS-Supplementary Information

Jian Cui[1] , Xuepo Ma[1] , Long Chen[1] and Jianqiu Zhang*[1]

[1]Department of Electrical and Computer Engineering, the University of Texas at San Antonio, One UTSA Circle, San Antonio, TX 78249

Email: Jian Cui - cuijian1001@gmail.com; Xuepo Ma - maxuepo@gmail.com; Long Chen - becloned@gmail.com; Jianqiu Zhang*- michelle.zhang@utsa.edu;

*Corresponding author

Details of the simulation process of OpenMS

OpenMS(Version 1.7.0) is evaluated to have the best performance in [1]. In our comparison, we first created two .edta files [2] according to the required data format with five columns: RT, m/z, Intensity, charge and mymeda. In the first file of Q1, for every peptide in the testing set, we only listed the intervals that contain the retention time points reported by Tandem MS. In the second file of Q2, all candidate intervals for all testing peptides are listed. Subsequently, we convert the .edta files to the .featureXML files using the function FileConverter in OpenMS. Then we applied MapAligner to align the two featureXML files. In the last step, we used Featurelinker in OpenMS to find corresponding features. In this step, the parameter max_pair_distance has two fields that require user input values: RT and MZ. We set RT to two possible values 500 and 700, while MZ is set as 0.01. We tried different settings to ensure the best result. The processing procedure in OpenMS is shown in Figure 1. OpenMS can achieve a 79.79% and 80.35% accuracy under these settings which has little difference.

Example of an aligned peptide by SCFIA which is missed by Gwarping

In Figure 2, we can see that the XIC is very crowd in Q2 for the peptide K.VWLDPNETNEIANANSR.Q. The interfering feature in the Q2 is very close to the true time point mapped from Q1. The time difference between the interfering peak apex and the mapped time point is 131.7s, and the true corresponding feature has a larger time difference of 150.9s. Therefore, if we only correct the mean time shift, we can not identify the correct corresponding feature in Q2. However, the AR statistic between the true corresponding pair is

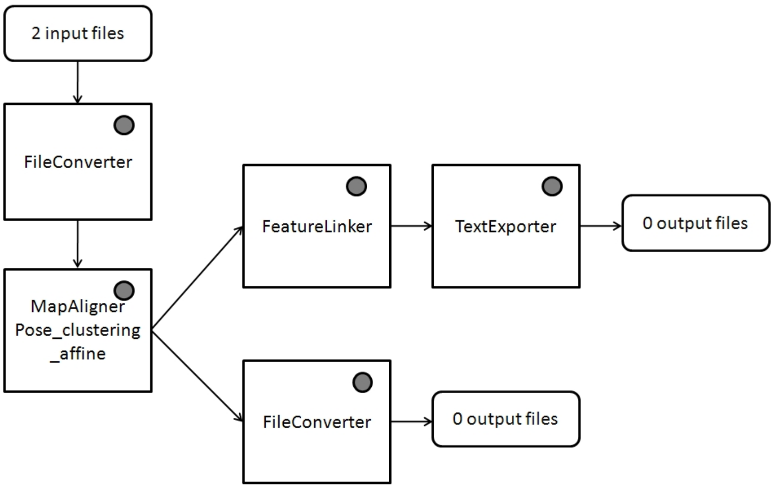

Figure 1: The simulation process for OpenMs.

0.8993, while the AR statistic between the feature of interest in Q1 and the interfering feature in Q2 is 0.6648. After calculating the total probability, the true corresponding feature pair provides the maximum likelihood probability, which finally leads to the correct identification.

Example of a peptide that is not aligned by SCFIA and Gwarping

In Figure 3, the time difference between the closest interfering feature in Q2 and the feature of interest in Q1 is 31.7s, with an AR score of 0.8999. The time shift between the corresponding feature as indicated by the "ground truth" is 195.7s with an AR score of 0.8751. So based on our probability model, the "interfering feature" has a higher probability of being the actual corresponding feature match. In this case, we suspect that the Tandem MS provided wrong identification.

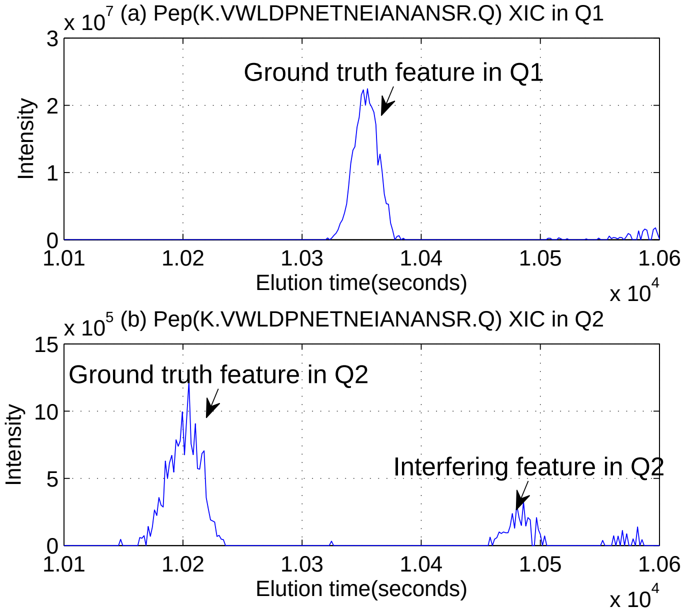

Figure 2: The XICs of the peptide sequence "K.VWLDPNETNEIANANSR.Q". in Q1 and Q2 after applying the Gwarping function.

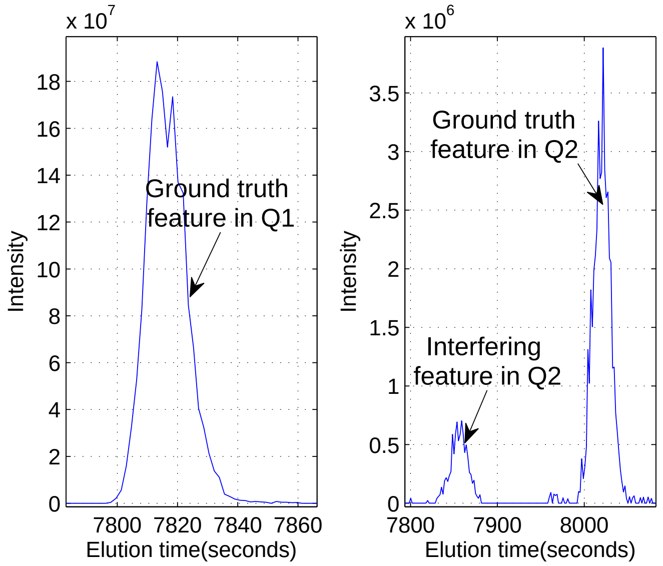

Figure 3: Zoomed in view of the XICs of "R.STSESTAALGC[160.03]LVK.D" in Q1 and Q2 after applying the Gwarping function.

References

1. Lange E, Tautenhahn R, Neumann S, Gröpl C: **Critical assessment of alignment procedures for LC-MS proteomics and metabolomics measurements**. *BMC bioinformatics* 2008, **9**:375.

2. Sturm M, Bertsch A, Gröpl C, Hildebrandt A, Hussong R, Lange E, Pfeifer N, Schulz-Trieglaff O, Zerck A, Reinert K, et al.: **OpenMS – An open-source software framework for mass spectrometry**. *BMC bioinformatics* 2008, **9**:163.
